# Wear Resistance of Ti–6Al–4V Alloy Ball Heads for Use in Implants

**DOI:** 10.3390/jfb12040065

**Published:** 2021-11-24

**Authors:** Svetlana Skvortsova, Aleksei Orlov, Georgii Valyano, Victor Spektor, Natalia Mamontova

**Affiliations:** 1 Moscow Aviation Institute, National Research University, 4, Volokolamskoe Highway, 125993 Moscow, Russia; skvortsova11@gmail.com (S.S.); spektorvs@gmail.com (V.S.); mamontova.natalie@gmail.com (N.M.); 2 Joint Institute for High Temperatures of Russian Academy of Sciences (JIHT), 13 Bd.2, Izhorskaya Str., 125412 Moscow, Russia; gvalyano@yandex.ru

**Keywords:** titanium alloy, ball head, hip joint endoprosthesis, modified surface, vacuum ion–plasma nitriding, microhardness, structure, torque, coefficient of friction, titanium nitride

## Abstract

The effect of thermohydrogen treatment and vacuum ion–plasma nitriding on the determination of the volume and surface structure of ball heads made of Ti–6Al–4V alloy was studied. It was found that the submicrocrystalline structure formed in the head during thermohydrogen treatment makes it possible to achieve hardness values of 39–41 units HRC and a surface roughness of 0.02 μm. It was shown that the creation of a modified layer consisting of ε (TiN) and δ (Ti_2_N) titanium nitrides on the surface of a ball head and the solid interstitial solution of nitrogen in α-titanium makes it possible to completely eliminate material wear when testing for friction on ultra-high-molecular-weight polyethylene. The equivalent analysis was also conducted with a ball head that had been implanted in a human body for 12 years. It was found that the change in the color of the head, from slightly golden after nitriding to metallic, is due to the formation of an oxynitride nanoscale layer on the surface. It was shown that in contrast with films made of titanium oxide, the film developed in this study has high wear resistance.

## 1. Introduction

In recent years, titanium and its alloys have increasingly been used not only in aerospace but also in other sectors of the world economy. Titanium and titanium-based alloys are becoming the main material for the manufacture of medical implants and surgical instruments, replacing stainless steels and cobalt–chromium alloys [1,2,3].

Titanium alloys used for medical purposes (Ti–6Al–4V, Ti–6Al–7Nb) with a sufficiently high strength (σ_B_ > 900 MPa) have high corrosion resistance in the biological environment. This is due to the existence of a nanometer-thick oxide film on their surface that provides corrosion resistance and prevents the component ions from releasing into the surrounding tissues after a titanium implant is installed in the human body, and additionally, they have good biocompatibility [4,5].

However, the adhesive strength of the protective oxide film with titanium is low. Therefore, it peels off from the titanium base due to the exploitation of implants under the action of mechanical contact stresses. This leads to intensive wear of the titanium components. In particular, catastrophic wear is observed in the titanium implant surface if cyclic contact stresses act in a biological environment [6,7,8,9,10,11,12]. This often leads to premature failure of implants, and reoperation is then required.

The ball head is one of the main components of a hip joint endoprosthesis and it is subject to mechanical contact stresses. To prevent wear, it is most often made of ceramics, which significantly increases the cost compared to endoprostheses made of stainless steels or cobalt–chromium alloys. However, when dissimilar metals are used, a galvanic pair is formed, which leads to contact corrosion [13,14,15,16,17].

To increase the wear resistance of titanium alloys, nitrogen is most often used, both in the creation of nitrogen-containing “alpha” layers on the surface and in its application as nitride coatings. This is achieved by using thermal diffusion saturation with nitrogen from the gaseous environment, by ion implantation, or by applying PVD coatings [18,19,20,21,22,23,24,25]. The disadvantages of these technologies include:

1. A change in the volume structure of a device during thermal diffusion saturation at elevated temperatures, which leads to a significant deterioration in the mechanical properties [18,19,20];
2. A change in the surface microgeometry after nitriding at elevated temperatures, which excludes the use of such processing as a way of applying a finish to the completed product because one of the main conditions for the operation of friction components is a high level of surface cleanliness [21,23,24];
3. The low adhesion strength of nitride coatings, which quite often leads to their peeling off during use [22]. In addition, the existence of a very hard nitride coating on the “soft” titanium base causes the appearance of an “eggshell” effect, i.e., cracking of the coating during use;
4. The formation of a “droplet phase” on the surface of the product during the formation of a nitride coating, which significantly increases its roughness [26].

Despite the fact that the Ti–6Al–4V and Ti–6Al–7Nb alloys belong to the (α + β)-class, they contain no more than 15% of the β-phase when in an equilibrium state. This does not allow them to be significantly strengthened by classical heat treatment. Increasing hardness is necessary to improve the surface cleanliness during polishing and to prevent the occurrence of the “eggshell” effect [27]. However, titanium alloys with the desired structure and properties can be prepared using thermohydrogen treatment (THT) [28,29]. THT is a combination of reversible hydrogen alloying with thermal action on a hydrogenated material. The technological schemes of THT include three main elements: saturation with hydrogen, thermal effect on the hydrogen-alloyed material, and dehydrogenation in vacuum. The use of such treatment can significantly improve the properties of titanium alloys [29].

One of the advanced technologies for the surface-hardening of titanium alloys is low-temperature vacuum ion–plasma nitriding [26]. This allows surface nitrogenation of the of completed product to be carried out at temperatures not exceeding 600 °C and with short holding times (up to 1 h). This excludes the change in both the surface microgeometry and the volume structure formed in the previous technological stages. Thus, a new material is created based on the titanium alloy Ti–6Al–4V with a gradient surface structure [30].

In this study, we characterized the effect of thermohydrogen treatment and low-temperature vacuum ion–plasma nitriding on the determination of the volume and surface structure of titanium ball heads after production as well as after use in a human body for 12 years.

## 2. Materials and Methods

The studies were carried out on ball heads made of a hot-rolled Ti–6Al–4V alloy rod, the chemical composition of which is shown in Table 1.

Hydrogenation annealing to a concentration of 0.8 wt.% was carried out in a high-frequency hydrogen atmosphere at a temperature of 850 °C. Vacuum annealing was carried out in the VEGA-3M vacuum furnace.

Ion–vacuum nitriding was carried out at a temperature of 550 °C and holding time of 40 min in a gas mixture of 15% N and 85% Ar. Before releasing gases in the chamber at a vacuum no higher than 3 × 10^−3^ Pa, ion etching was carried out to remove the oxide film and other contaminants.

The depth of the nitrogen-hardened surface layer was determined using the oblique section method. The microhardness was measured on a Micromet 5101 device using the Vickers method with a load of 0.5 N, in accordance with GOST 9450 using the NEXSYS ImageExpert MicroHardness 2 software package with a step of 50 μm.

The microhardness was measured on five nitrided ball heads in three fields of view (a total of 15 measurements for each measurement step). Similar studies using five fields of view were carried out on a single ball head that had been implanted for 12 years in a human body.

The roughness of the surface was determined using a Hommel Tester T500 profilometer, in accordance with GOST 2789.

Electron microscopic analysis of foils was carried out on the JEM-200C transmission electron microscope with an accelerating voltage of 125 kV.

Metallographic studies were carried out on the AXIO Observer.A1m optical microscope (Carl Zeiss Meditec AG, Jena, Germany) using the bright field method in air. The images were analyzed using the NEXSYS ImageExpert Pro3 software package.

X-ray phase analysis at room temperature was carried out on a DRON-7 diffractometer with a quasi-focusing Bragg–Brentano scheme at an accelerating voltage of 35 kV and an anode current of 25 mA in filtered copper (CuKα) radiation.

Micro X-ray spectral analysis was performed on the Nova NanoSem 650 scanning electron microscope according to the standardless method using the EDAX energy-dispersive analysis system.

The nitrogen content was measured in five fields of view on two different ball heads: directly after nitriding and after 12 years of implantation in a human body.

The determination of the work durability of a friction pair, ball head–ultra-high-molecular-weight polyethylene (UHMWPE), was carried out in the absence of lubrication with a vertical load on the femoral head of 2250 N for 300 complete cycles of rotation of the head at a frequency of 1 Hz (GOST R 52,640) on the universal testing machine LFM-503. The tests were carried out in “harsh” conditions to ensure the high workability and reliability of the component in the human body: (i) the load was 3 times the average weight of a person; (ii) dry friction, because it is still not completely clear whether the artificial joint works under dry friction conditions or in the presence of joint fluid, as in a healthy joint; and (iii) 300 rotations correspond to at least 10 years of endoprosthesis implantation in the human body [31,32].

The experimental results were subjected to statistical processing using the Statistica software package. The sample mean, sample variance, standard deviation, confidence interval of the average value and measurement error were determined with a probability of 0.95.

## 3. Results

To increase the wear resistance of titanium ball heads, it is necessary to increase their hardness to 40–42 units HRC. This ensures the required surface finish (Ra) during the polishing process (not more than 0.03 microns). Then, it is necessary to create a surface-modified layer that is firmly connected to its titanium base.

**Figure 1:**
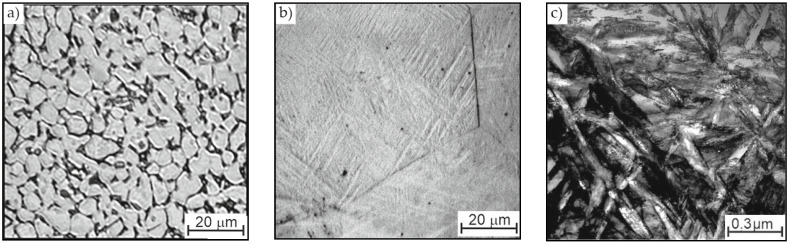
The structure of a ball heads in (**a**) the annealed state and after (**b**) hydrogenating annealing or (**c**) vacuum annealing.

High hardness of the ball heads was achieved using thermohydrogen treatment. The ball heads produced from an annealed rod had an equilibrium structure, with a globular morphology of α-phase particles and a hardness of 30–32 units HRC (Figure 1a). The introduction of 0.8 wt. % hydrogen into ball heads allows an almost single-phase β-structure to be obtained after cooling to room temperature (Figure 1b). During subsequent degassing under vacuum, the β-phase becomes unstable and a β → α transformation is initiated. The low temperature of vacuum annealing (625 °C) ensures the formation of a dispersed structure in heads, with a hardness of 39–41 units HRC (Figure 1c).

The Ti–6Al–4V alloys, when THT-processed, have high strength (1200 MPa) and low plasticity (≈3.5%). However, the plasticity value is satisfactory since the head of the endoprosthesis is a structural element of the hip joint and does not experience loads that require a high reserve of plasticity.

The high-hardness heads were polished to a surface finish of R*_a_* = 0.02 µm and subjected to vacuum ion–plasma nitriding at a temperature of 550 °C. The total process time did not exceed 40 min. As the temperature of the finishing treatment was lower than the vacuum annealing temperature, no significant changes were observed in the volume structure of the ball heads, and the surface finish decreased by only 0.01 microns.

**Figure 2:**
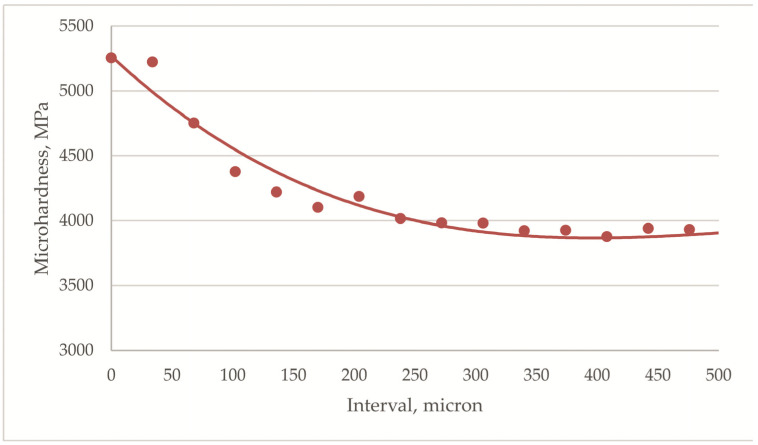
Depth distribution of microhardness of nitrided ball heads made of Ti–6Al–4V alloy (each experimental point is an average value based on the results of three measurements on five heads).

The depth of the nitrogen-hardened modified surface layer on the ball heads was determined. The surface hardness was measured at 5200 MPa. At some distance from the surface, the hardness decreases monotonically, and at a distance of 150 μm, the hardness reaches 3900 MPa and then barely changes (Figure 2). Thus, the depth of the nitrogen-modified layer was 150 μm.

**Figure 3:**
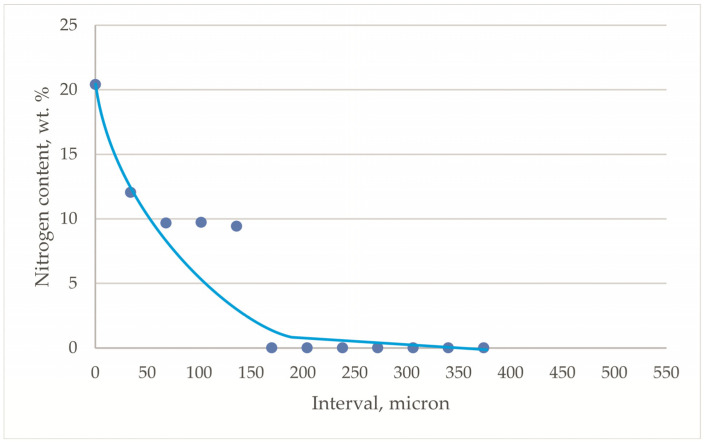
Depth distribution of nitrogen content of nitrided ball heads made of Ti–6Al–4V alloy (each experimental point is the average value of the results of five measurements on one head).

The chemical composition of the ball head was determined at approximately the same points as where the microhardness measurements were carried out. It was found that the ball head surface contained about 20 mass. % nitrogen, which gradually decreased with increasing depth, and the value at a distance of 150 μm corresponded to the average content in the alloy (Figure 3). Thus, previous studies have shown that the high microhardness of the ball heads’ surface layers is due to the increased nitrogen content.

**Figure 4:**
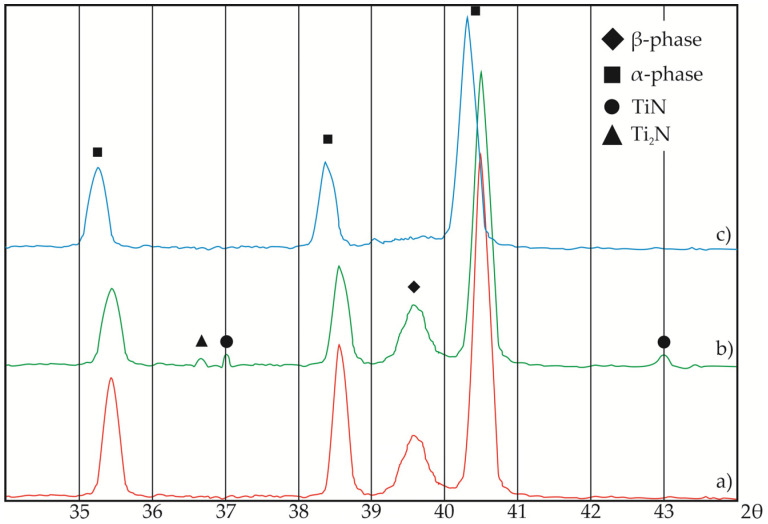
The X-ray diffraction of the ball heads (**a**) in the annealed state and after (**b**) THT and nitriding or (**c**) 12 years of implantation in a human body.

X-ray diffraction analysis showed that δ (TiN) and ε (Ti_2_N) titanium nitrides are formed in the near-surface layers of the ball heads after vacuum ion–plasma nitriding. The periods of the crystal lattice of the α-phase remain practically unchanged in comparison with the annealed state (Figure 4a,b).

**Figure 5:**
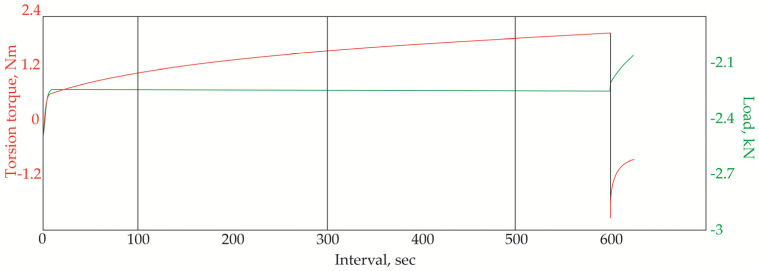
The torque and load during testing of the friction pair Ti–6Al–4V alloy ball head with modified surface—UHMWPE insert.

In the next stage, we determined the wear resistance of the titanium ball heads after nitriding. The torque values in a friction pair of Ti–6Al–4V ball head–UHMWPE insert were measured under the conditions of absence of lubrication, constant axial load of 2250 N, and rotational speed of 0.5 rev/s. The friction coefficient was calculated according to the GOST R 52,640 method, and a visual inspection was conducted of the friction pair (Figure 5).

**Figure 6:**
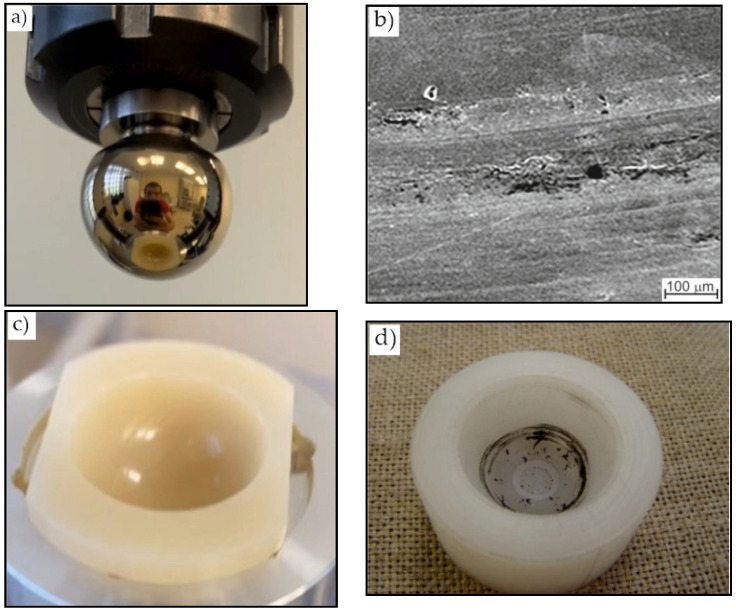
The appearance of (**a**,**b**) ball heads and (**c**,**d**) UHMWPE inserts with (**a**,**c**) modified and (**b**,**d**) unmodified surface after wear tests.

The average torque during the tests was within 1.0 and 1.8 Nm; the friction coefficient was 0.04. There were no signs of wear on the friction pair components (Figure 6a,c).

In accordance with GOST R 52,640, if the head can withstand 300 revolutions, the durability of the friction unit is ensured for at least 10 years.

For comparison, a ball head was tested without additional THT and surface treatment. During the test, the temperature was increased up to 75 °C in the contact zone, and an increase in the friction coefficient from 0.03 to 0.13 was observed. After the experiment, traces of wear were clearly visible on the surface of the head and wear products in the insert (Figure 6b,d).

Thus, these studies show that the surface modification of titanium ball heads makes it possible to eliminate their wear during friction with a UHWMPE insert.

**Figure 7:**
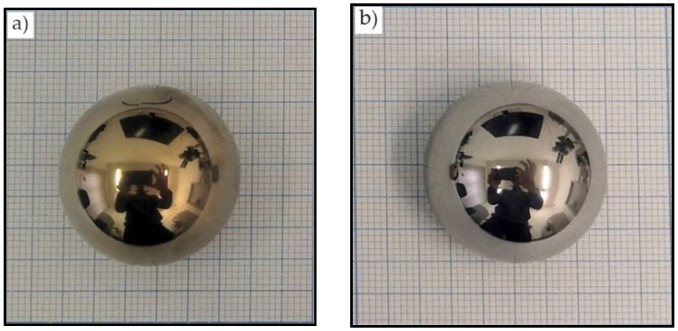
The appearance of the ball heads after (**a**) nitriding and (**b**) removal from a human body after 12 years of implantation.

In the next stage, the ball head was examined after its removal from a person after 12 years of implantation. In Russia, during revision operations, the removed components are either disposed of in the hospital or given to the manufacturer for research; this procedure does not require the patient’s ethical approval. The devices were provided to us by the manufacturer company. No signs of wear in the head and insert were detected by visual inspection. However, a change in the color of the head was detected. After nitriding, the ball heads had a golden hue (Figure 7a), but the removed head had a metallic hue (Figure 7b).

It should be noted that no change was observed in the color and chemical composition of the head surface under an air atmosphere following 20 years of observation.

**Figure 8:**
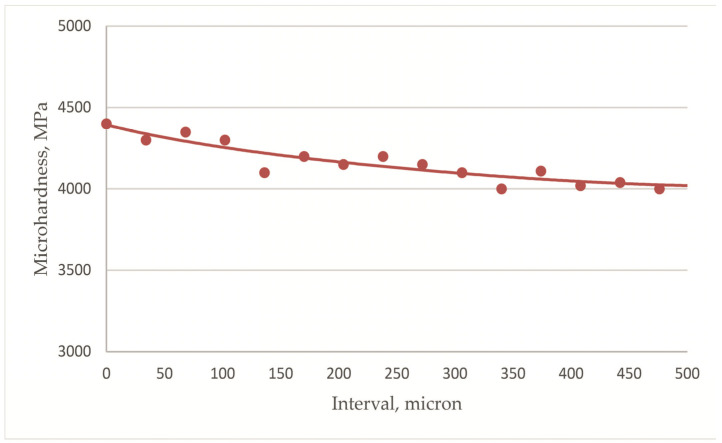
Depth distribution of microhardness of a nitrided ball head after 12 years of implantation in a human body (each experimental point is an average value based on the results of 5 measurements on one head).

The micro-X-ray spectral analysis of the ball head surface that was conducted showed that the amount of nitrogen decreased to 7.8 mass. %, i.e., it decreased by 2.5 times (Figure 3). Oxygen was present in the amount of 4.1 mass. %. No ε (Ti_2_N) and δ (TiN) titanium nitrides reflections were observed in the diffractograms (Figure 4c). Period *a* of the α-phase crystal lattice increased from 0.2926 (after nitriding) to 0.2938 nm (after operation). A decrease in the nitrogen content on the surface led to a decrease in surface microhardness to 4400 MPa, but the further decrease in hardness deeper into the head was more gradual, and the depth of this layer increased almost two-fold (Figure 8). Thus, the redistribution of nitrogen and the formation of a more extended interstitial solid solution of nitrogen in α-titanium occurs during its use in a biological environment.

To characterize the wear resistance of the head, the torque was measured in a friction pair with a UHWMPE insert.

**Figure 9:**
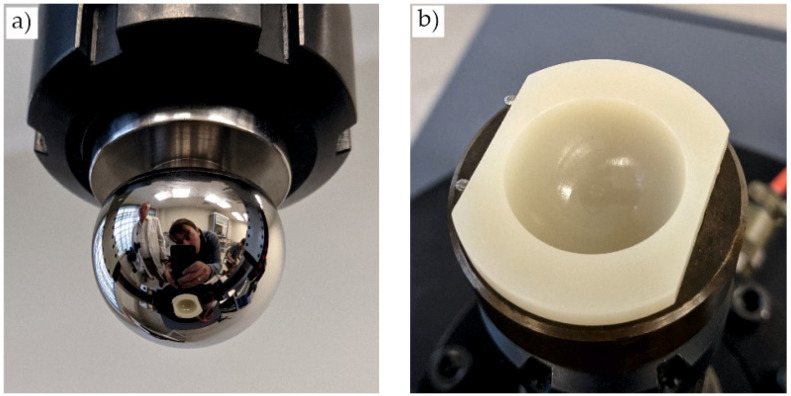
The appearance of (**a**) the removed ball head and (**b**) the UHMWPE inserts after wear testing.

The average torque was in the range of 1.3–2.0 Nm, which is slightly higher than the values for the ball head after nitriding (Figure 5). The value of the friction coefficient increased from 0.04 to 0.05. However, most importantly, component wear was not observed (Figure 9). Thus, the total uptime of the ball heads made of titanium alloy Ti–6Al–4V is potentially longer than 30 years.

Apparently, the changes in the chemical composition and the color of the titanium ball heads during long-term operation in a biologically active environment are associated with the activation of the oxygen adsorption process. This is due to the higher affinity of titanium to oxygen than to nitrogen and the higher stability of titanium oxides [33]. Oxygen adsorption on the surface is the reason for the gradual dissolution of ε (Ti_2_N) and δ (TiN) titanium nitrides, and the diffusion of nitrogen deeper into the device. An oxynitride film of nanoscale thickness is slowly formed on the surface. Unlike the oxide film, it has high adhesive strength with titanium and does not peel off under the action of mechanical contact stresses.

## 4. Conclusions

Studies have shown that Ti–6Al–4V alloy can be used in the production of endoprosthesis elements operating under the action of mechanical contact stresses. To ensure their high wear resistance, high surface cleanliness is necessary. This can be achieved through the formation of a submicrocrystalline structure with a hardness of 39–41 units HRC under thermohydrogen treatment. It is also necessary to create a thin film with high adhesive strength on the titanium base that does not peel off under the action of mechanical contact stresses. This can be achieved by low-temperature vacuum ion–plasma nitriding. A modified surface was formed with a depth of at least 150 microns, containing titanium nitrides ε (Ti_2_N) and δ (TiN) and an interstitial solid solution of nitrogen in α-titanium. Long-term operation in a biological environment leads to a change in the chemical composition of the titanium ball head surface. An oxynitride film of nanoscale thickness is then formed instead of titanium nitrides. Nevertheless, it remains firmly connected to the titanium base, and the ball heads retain high wear resistance. The tests estimated that the approximate service life of ball heads made of titanium alloy was at least 30 years.

## Figures and Tables

**Table 1:** Chemical composition of Ti–6Al–4V alloy.

Alloy	Basic Alloying Elements, (wt. %)			Impurities, (wt. %)				
	Ti	Al	V	Fe	C	O	N	H
Ti–6Al–4V	Balance	5.8	4.1	0.25	0.02	0.11	0.01	0.008
ISO 5232-3	Balance	5.5–6.75	3.5–4.5	≤0.3	≤0.08	≤0.2	≤0.05	≤0.015
